# Delineating the autistic phenotype in children with neurofibromatosis type 1

**DOI:** 10.1186/s13229-021-00481-3

**Published:** 2022-01-04

**Authors:** Anita K. Chisholm, Kristina M. Haebich, Natalie A. Pride, Karin S. Walsh, Francesca Lami, Alex Ure, Tiba Maloof, Amanda Brignell, Melissa Rouel, Yael Granader, Alice Maier, Belinda Barton, Hayley Darke, Gabriel Dabscheck, Vicki A. Anderson, Katrina Williams, Kathryn N. North, Jonathan M. Payne

**Affiliations:** 1 Murdoch Children’s Research Institute, 50 Flemington Road, Parkville, VIC 3052 Australia; 2 Department of Paediatrics, Faculty of Medicine, Dentistry and Health Sciences, University of Melbourne, Parkville, VIC 3010 Australia; 3 The Royal Children’s Hospital, 50 Flemington Road, Parkville, VIC 3052 Australia; 4 Kids Neuroscience Centre, The Children’s Hospital at Westmead, 178A Hawkesbury Road, Westmead, NSW 2145 Australia; 5 Center for Neuroscience and Behavioral Medicine, Children’s National Hospital, Michigan Avenue NW, Washington, DC 20310 USA; 6 Department of Paediatrics, School of Clinical Sciences, Monash University, 246 Clayton Road, Clayton, VIC 3168 Australia; 7 Developmental Paediatrics, Monash Children’s Hospital, 246 Clayton Road, Clayton, VIC 3168 Australia; 8 Sydney Medical School, University of Sydney, Camperdown, NSW 2050 Australia; 9 Children’s Hospital Education Research Institute, The Children’s Hospital at Westmead, 178A Hawkesbury Road, Westmead, NSW 2145 Australia

**Keywords:** Autism, Autistic behaviours, Autism Diagnostic Interview-Revised (ADI-R), Autism Diagnostic Observation Schedule-Second Edition (ADOS-2), Neurofibromatosis type 1

## Abstract

**Background:**

Existing research has demonstrated elevated autistic behaviours in children with neurofibromatosis type 1 (NF1), but the autistic phenotype and its relationship to other neurodevelopmental manifestations of NF1 remains unclear. To address this gap, we performed detailed characterisation of autistic behaviours in children with NF1 and investigated their association with other common NF1 child characteristics.

**Methods:**

Participants were drawn from a larger cross-sectional study examining autism in children with NF1. The population analysed in this study scored above threshold on the Social Responsiveness Scale-Second Edition (T-score ≥ 60; 51% larger cohort) and completed the Autism Diagnostic Interview-Revised (ADI-R) and/or the Autism Diagnostic Observation Schedule-Second Edition (ADOS-2). All participants underwent evaluation of their intellectual function, and behavioural data were collected via parent questionnaires.

**Results:**

The study cohort comprised 68 children (3–15 years). Sixty-three per cent met the ADOS-2 ‘autism spectrum’ cut-off, and 34% exceeded the more stringent threshold for ‘autistic disorder’ on the ADI-R. Social communication symptoms were common and wide-ranging, while restricted and repetitive behaviours (RRBs) were most commonly characterised by ‘insistence on sameness’ (IS) behaviours such as circumscribed interests and difficulties with minor changes. Autistic behaviours were weakly correlated with hyperactive/impulsive attention deficit hyperactivity disorder (ADHD) symptoms but not with inattentive ADHD or other behavioural characteristics. Language and verbal IQ were weakly related to social communication behaviours but not to RRBs.

**Limitations:**

Lack of genetic validation of NF1, no clinical diagnosis of autism, and a retrospective assessment of autistic behaviours in early childhood.

**Conclusions:**

Findings provide strong support for elevated autistic behaviours in children with NF1. While these behaviours were relatively independent of other NF1 comorbidities, the importance of taking broader child characteristics into consideration when interpreting data from autism-specific measures in this population is highlighted. Social communication deficits appear similar to those observed in idiopathic autism and are coupled with a unique RRB profile comprising prominent IS behaviours. This autistic phenotype and its relationship to common NF1 comorbidities such as anxiety and executive dysfunction will be important to examine in future research. Current findings have important implications for the early identification of autism in NF1 and clinical management.

**Supplementary Information:**

The online version contains supplementary material available at 10.1186/s13229-021-00481-3.

## Background

Autism spectrum disorder (hereafter referred to as autism) is a neurodevelopmental disorder defined by differences in social communication and interaction, as well as the presence of restricted interests and repetitive behaviours that create limitations to activities and participation [1]. Consistent with current conceptualisation of autism as a ‘spectrum’ [2, 3], substantial variability is evident in its clinical characteristics [4]. Autistic individuals present with a wide range of cognitive and language abilities [5, 6], and commonly co-occurring difficulties such as attention deficit hyperactivity disorder (ADHD) and generalised anxiety disorder [7] further increase the phenotypic complexity of autism. This clinical heterogeneity is mirrored by a highly diverse genetic architecture. Large cohort studies have identified hundreds of autism susceptibility genes, comprising both common variants with low penetrance and rare variants with higher penetrance [8–11]. Despite ongoing advances in understanding how genes contribute to the development of autism, the neurobiological aetiology remains unknown in most affected individuals [12]. Moreover, the marked genetic and clinical complexity of autism has impeded the establishment of clear genotype–phenotype associations and the development of effective treatments [13, 14].

More recently, interest has turned to the investigation of syndromic forms of autism as promising candidates for elucidating the causal pathways to autism [14]. Neurofibromatosis type 1 (NF1) is a common monogenic syndrome, with a birth incidence of 1 in 2700 [15], which offers unique opportunities to systematically examine the autistic phenotype in the context of more homogeneous genetic aetiology. At the molecular level, mutations in the *NF1* gene lead to hyperactivation of the Ras-mitogen-activated protein kinase (MAPK) pathway as well as disinhibition of mechanistic target of rapamycin (mTOR) kinase signalling [16], both of which have been implicated in the pathogenesis of autism [17, 18]. Importantly, these signalling pathways offer potential molecular targets for disease-directed pharmacological treatments in NF1 that have, so far, been elusive for idiopathic autism [19, 20]. At the clinical level, a growing body of literature has indicated an elevated autistic trait burden in NF1 [18–26], with recent meta-analytic evidence demonstrating a large effect size (*g* = 0.91) of autistic behaviours on parent-rated trait-based questionnaires [27]. Studies employing well-established diagnostic instruments have also suggested that autism prevalence in children with NF1 ranges between 11 and 26% [23, 28, 29], significantly higher than the 1–4% prevalence reported in the general population [30, 31].

As a single-gene condition with high autism penetrance, NF1 presents a valuable genetic model for advancing our understanding of the neurobiological mechanisms of autism. However, detailed characterisation of the expression of autistic behaviours in children with NF1 has been limited. Increasing our understanding of the autistic phenotype in this condition is not only important for identifying the most suitable clinical outcomes in future clinical trials, but also for the goal of establishing precise genotype–phenotype relationships. To date, three studies have attempted to quantify autistic behaviours and diagnostic rates in NF1 using the ‘gold standard’ [32] Autism Diagnostic Observation Schedule-Second Edition (ADOS-2) [33] and/or the Autism Diagnostic Interview-Revised (ADI-R) [34]. Both the ADOS-2 observational assessment and the ADI-R parent interview comprise a range of items relating to social communication skills and restricted and repetitive behaviours (RRBs); however, some of the behaviours sampled vary across the two measures. For each measure, specified individual behaviour ratings, referred to as algorithm items, are summed into subscales and/or domain scores to provide cut-off scores for autism. Garg et al. [35] compared ADOS-2 scores of children with NF1, who scored in the clinical range on the Social Responsiveness Scale-Second Edition (SRS-2; T-score ≥ 60) and met cut-off for ‘autism spectrum’ on the ADOS-2, to normative data of children with autism, as reported in the ADOS-2 manual [33]. While the autistic behaviour profiles were generally similar, the NF1 group with a research-based diagnosis of autism displayed relatively better eye contact, superior language skills, and fewer RRBs than the idiopathic autism group. Another study [36] reporting on select ADI-R and ADOS-2 outcomes in children with NF1 who scored in the clinical range on the SRS-2 found mean scores for males and females on the ‘stereotyped and repetitive motor mannerisms’ ADI-R subscale to be lower relative to other ADI-R RRB subscales. However, since this study did not present the item-level data needed to understand specific autistic behaviours, the NF1 profile of RRB behaviours was not clear. More recently, Geoffray and colleagues [37] performed cross-syndrome comparisons of children who met cut-off scores on the ADOS-2 and ADI-R across NF1, Noonan syndrome, and cardiofaciocutaneous syndrome, related genetic conditions involving dysregulation of the Ras/MAPK pathway (RASopathies) [38, 39]. Subtle differences in behaviour profiles were reported between the groups, with greater social communication impairments but fewer RRBs in children with NF1 as compared to the other RASopathies. However, the evaluation of RRBs was restricted to ADI-R composite scores and the ADOS-2, which does not provide a comprehensive assessment of these behaviours [40]. This information fails to capture the full spectrum of the RRB domain as defined by DSM-5 [1], and the composite ADI-R scores potentially obscure meaningful individual differences in RRB behaviours.

Despite mounting evidence that NF1 is associated with autism [23, 28, 29], there is still active debate in the literature regarding the potentially confounding effects of the broader neurodevelopmental manifestations of NF1 on the measurement of autistic behaviours [41–44]. Some have argued that higher scores on the SRS-2 are attributable to common NF1 comorbidities of ADHD, internalising symptoms, and language problems and that such data fail to provide evidence of an increased risk for autism [41–43]. While the frequent co-occurrence of autism with other neurodevelopmental disorders is well established [45] and formally recognised by DSM-5 criteria [1], the careful examination of associations between autistic behaviours and other NF1 characteristics is essential for improving diagnostic accuracy in NF1. To date, these relationships have not been investigated in NF1 using gold standard autism-specific measures.

The primary aim of the current study was thus to perform detailed characterisation of the autistic phenotype in children with NF1. We conducted item- and domain-level analyses of core autistic behaviours using the ADI-R and ADOS-2 in a ‘screen positive’ NF1 cohort that scored in the clinical range on the parent-rated SRS-2 (T-score ≥ 60). This screening tool has been demonstrated to have high sensitivity (e.g. 91% for parent report) for identifying autistic children [46]. Since the ADI-R and ADOS-2 algorithm RRB items (i.e. a specified subset of items that contribute to diagnostic classification) do not capture the full range of these behaviours as defined by DSM-5, we utilised all RRB items to comprehensively assess these autism criteria. We chose a screen-positive NF1 sample for two reasons. First, existing research has demonstrated the intrinsically continuous nature of autistic behaviours in NF1 and the general population [3, 21, 26], and not limiting our sample to those who met threshold for the categorical diagnosis allowed for examination of the broader range of autistic and associated characteristics in children with NF1 who are ‘at risk’ of autism. Second, from a therapeutic perspective, children with social communication difficulties and/or RRBs may benefit from early intervention regardless of whether their difficulties fulfil formal diagnostic criteria [47]. In view of commonly co-occurring cognitive, language, and behavioural difficulties in NF1 [48, 49], and their symptom overlap with autism [50, 51], our secondary aim was to explore the associations between NF1-related neurodevelopmental comorbidities and autistic behaviours in our screen-positive NF1 cohort.

## Methods

### Participants

**Fig. 1:**
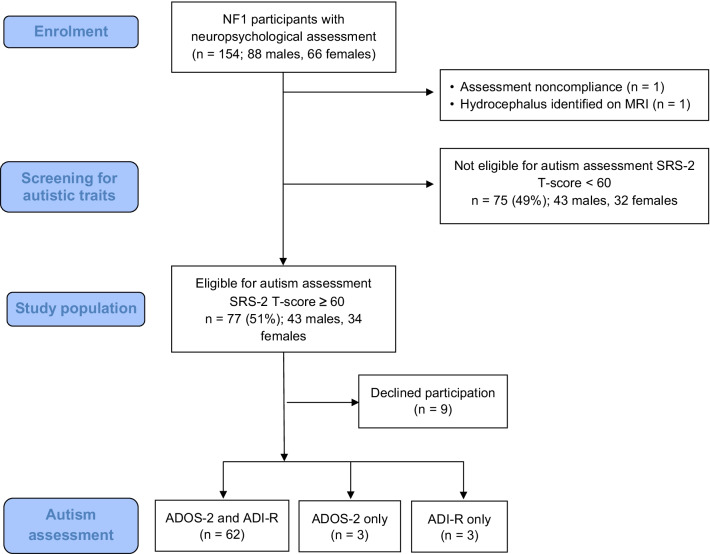
Flow diagram depicting enrolment and assessment process of participants with NF1

Participants were a subset of children enrolled in a larger prospective cross-sectional study investigating the autistic phenotype and social functioning in children with NF1 [52]. As part of the larger study, children were sequentially recruited from NF1 clinics at three centres: (1) The Royal Children’s Hospital/Murdoch Children’s Research Institute, Melbourne, Australia; (2) The Children’s Hospital at Westmead, Sydney, Australia; and (3) the Children’s National Hospital, Washington DC, USA. All participants were diagnosed clinically with NF1 according to National Institutes of Health criteria [53]. Genetic confirmation was not available for the majority of the cohort. Participants were aged between 3 and 15 years at the time of the initial cognitive assessment. Exclusion criteria were: symptomatic intracranial pathology (other than asymptomatic gliomas), deafness, severely impaired vision, and insufficient English to complete assessments. Participants from the larger cohort were included in the current study if they scored in the clinical range on the parent-rated SRS-2 autism trait questionnaire (T-score ≥ 60), and underwent detailed evaluation for autistic behaviours using the ADOS-2 [33] and/or ADI-R [34]. Fifty-one per cent of the larger cohort were eligible for in-depth autism assessment, resulting in a final study sample of 68 children with NF1 (38 males, 30 females; see Fig. 1).

### Direct assessments

#### Autism Diagnostic Interview-Revised (ADI-R)

The ADI-R [34] is a semi-structured, standardised parent interview designed to identify core autistic behaviours. It is composed of three functional domains: Qualitative Abnormalities in Reciprocal Social Interaction (Social-ADI); Qualitative Abnormalities in Communication (Communication-ADI); and Restricted, Repetitive, and Stereotyped Patterns of Behavior (RRB-ADI). Individual items concerning autistic behaviours have two ratings: ‘current’ behaviours occurring in the three months preceding the interview; and ‘lifetime’ ratings which are used to calculate the diagnostic algorithms. Lifetime ratings reflect specified behaviours at ages 4.0–5.0 years or the most abnormal behaviours to have ‘ever’ occurred in the child’s lifetime (for participants aged < 4.0 years, current/ever ratings are used). We report lifetime ratings when examining the NF1 autistic phenotype but use current ADI-R ratings when exploring associations with parent-reported psychopathology and IQ to ensure contemporaneous developmental periods are compared. Items were coded according to the examiner’s judgement of the presence or extent of a specified behaviour using an ordinal scale from 0 (absent) to 3 (present in extreme form). As per ADI-R scoring conventions, all scores of 3 were converted to 2 for the calculation of diagnostic algorithm scores. In order to meet the ADI-R classification criteria for autism, cut-offs in all three domains must be exceeded. It should be noted, however, that ADI-R cut-offs were constructed to diagnose ‘autistic disorder’ as defined by DSM-4-TR criteria [54] and are less sensitive to the broader ‘autism spectrum disorder’ presentations captured by DSM-5 diagnostic criteria [1].

#### Autism Diagnostic Observation Schedule-Second Edition (ADOS-2)

The ADOS-2 [33] is a standardised, observational evaluation of the presence and severity of social communication deficits and RRBs. The current study employed either Module 2, appropriate for children capable of flexible phrase speech (*n* = 9), or Module 3, for verbally fluent participants (*n* = 56). Each item was scored on a scale from 0 to 3 with higher scores indicating greater impairment in behaviours. The Overall raw score was calculated by converting scores of 3 to 2 and summing scores from a specified subset of items. This raw score forms the ADOS-2 diagnostic algorithm, which yields an empirically derived cut-off point for ‘autism spectrum’. We also calculated 10-point calibrated severity scores (CSS) for the domains of Social Affect (SA-CSS) and RRB (RRB-CSS), as well as for the combined Overall score (Overall-CSS) [40, 55]. These comparison scores were developed within specific ADOS-2 module groupings and are less influenced by chronological age and language than raw scores [56, 57].

#### Wechsler Preschool and Primary Scale for Children-Fourth Edition (WPPSI-IV) / Wechsler Intelligence Scale for Children-Fifth Edition (WISC-V)

General intellectual functioning was assessed using the WPPSI-IV [58] for children aged 3–5 years, or the WISC-V [59] for children aged 6–15 years. All subtests required for calculation of Full-Scale IQ (FSIQ) and primary index scales were administered. The FSIQ, Verbal Comprehension Index (VCI), Fluid Reasoning Index (FRI), and Visuospatial Index (VSI) were used in this study, all of which have a mean (M) of 100 and a standard deviation (SD) of 15. Higher scores reflect better performance.

#### Language milestones

Language milestones were elicited during the ADI-R parent interview and classified as a categorical variable (not delayed/delayed). As per the ADI-R protocol [34], Word Delay was defined as first words after 24 months of age, and Phrase Delay was defined as first 2–3 word phrases occurring after 33 months of age.

#### Social risk

We assessed children’s social risk via maternal education level (high risk < 12 years of schooling; low risk ≥ 12 years of schooling), as this risk factor has been found to primarily explain socioeconomic effects on child behavioural outcomes [60].

### Parent rating scales

For all rating scales, sex-specific norms were used when generating standardised scores.

#### Social Responsiveness Scale-Second Edition (SRS-2)

The SRS-2 [61] is a 65-item measure designed to index the severity of autistic behaviours in the general population. We used the preschool form for children aged 3–4.5 years and the school-age form for participants older than 4.5 years. Both versions provide DSM-5 compatible subscales of Social Communication and Interaction and Restricted Interests and Repetitive Behavior [1]. Higher scores are associated with more severe symptoms, and total T-scores ≥ 60 are considered to indicate impairment in reciprocal social behaviour (M = 50, SD = 10).

#### Conners ADHD DSM-IV Rating Scale (CADS)/Conners-Third Edition (Conners-3)

The CADS [62] and Conners-3 [63] were used as a dimensional index of ADHD symptom severity for participants aged 3–5 and 6–15 years, respectively. Both instruments provide T-scores for Inattentive and Hyperactive-Impulsive subscales (*M* = 50, SD = 10). Higher scores indicate more severe ADHD symptoms.

#### Child Behavior Checklist (CBCL)

The CBCL/1.5–5 [64] and CBCL/6–18 [65] were administered to determine the behavioural and emotional functioning of participants aged 3–5 and 6–15 years, respectively. For both versions of the CBCL, we used the DSM-oriented subscales of anxiety problems, affective problems, and oppositional defiant problems. The former two subscales were considered ‘purer’ measures of anxiety and depression for our sample, since the CBCL broadband internalising composite may be affected by the numerous somatic manifestations of NF1 [66]. All scales were converted into T-scores (*M* = 50, SD = 10) with higher scores indicating more severe psychopathology.

### Procedure

Ethical approval was received from the Human Research Ethics Committees of The Royal Children’s Hospital (HREC/16/RCHM/137), Sydney Children’s Hospitals Network (HREC/16/SCHN/42), and the Children’s National Hospital (Pro00007045). After explanation of the study at an initial clinical appointment, parents provided written, informed consent and completed questionnaires. Child cognitive assessments were administered by clinical neuropsychologists. If children were prescribed stimulant medication, they were requested to omit this a minimum of 24 h prior to evaluation. Participants who screened positive on the SRS-2 (T-score ≥ 60) were scheduled for ADOS-2 and ADI-R assessments, conducted by clinicians with established research reliability.

### Statistical analyses

IBM SPSS Statistics (version 27) was used for all statistical analyses. Data were checked for skewness and outliers to determine whether the assumption of normality was met; where data were not normally distributed, nonparametric tests were employed. Differences between the NF1 cohort and normative reference data were tested using one-sample *t* tests, with Cohen’s *d* indicating effect size (*d*, small: 0.2, medium: 0.5, large: 0.8) [67]. For comparisons between NF1 inheritance type groups (familial versus sporadic), we employed independent *t* tests and Mann–Whitney *U* tests for normally and non-normally distributed continuous variables, respectively. Chi-square tests were used to examine group differences for categorical variables. The false discovery rate (FDR) procedure was used to correct for multiple comparisons [68]. We examined ADOS-2- and ADI-R-derived autistic behaviours at both item and global levels. Item-level analyses are particularly informative since current diagnostic practice focuses on determining the presence or absence of specific autistic behaviours, and global scores may mask the identification of such characteristics. Items that were endorsed by more than half the sample (> 50%) were considered ‘common’. At the global level, we reported ADOS-2 and ADI-R algorithm, domain, and subscale scores and presented the proportions of participants who met standard algorithm cut-offs for these diagnostic instruments. While ADOS-2 global scores were not normally distributed, we reported means and 95% confidence intervals (CI) to provide comparability to relevant NF1 publications [28, 35, 37].

At an individual item level, we reported the frequency of endorsed autistic behaviours (nonzero codings) in each domain with bar graphs. To provide comprehensive information regarding the severity of symptom endorsement, we also reported the frequency of 1 vs. 2/3 codings and means and SDs at an item level for the ADI-R and ADOS-2. Bivariate correlational analyses investigated associations between autistic behaviours and other developmental comorbidities in NF1 (*r/rho*, 0.3: weak, 0.5: moderate, 0.7: strong) [69]. Since early language delay may be considered predictive of social communication deficits, linear regressions were conducted where significant associations between language delay and autistic behaviours were found.

## Results

### Sample characteristics

Clinical and demographic data for the larger cohort of 152 participants with NF1 are provided as supplementary data (Additional file 1: Table S1). Characteristics for the screen-positive NF1 sample analysed in this study are shown in Table 1. FSIQ and all reported Wechsler composite scores were significantly lower (> 1 SD) than the population mean, and 7.4% of the cohort had a clinical diagnosis of intellectual disability (ID) (i.e. evidence of intellectual and adaptive impairment during the developmental period) [1]. Parent report of early language development indicated that 25% of the sample exhibited single Word Delay and 39% exhibited Phrase Delay. While all participants scored above the SRS-2 cut-off on account of our sample selection criteria, considerable variability in severity of autistic behaviours was evident, with total T-scores ranging from 60 to 99. As expected, all SRS-2 T-scores were significantly elevated relative to population norms, as were scores reflecting internalising and externalising symptoms (all, *p* < 0.001), with Cohen’s *d* indicating large effect sizes (see Table 1). There was no effect of NF1 transmission (sporadic versus inherited) on social risk (*χ*^*2*^ = 0.95, *p* = 0.33), FSIQ, and Wechsler composite scores (all, *p* > 0.38), and SRS-2 total T-scores (*t* = 0.40, *p* = 0.69).

**Table 1:** Descriptive data for the NF1 sample (*N* = 68)^a^

NF1 characteristics		*p*	*d* (95% CI)
Age in years, M (SD) Range	9.0 (3.4) 3.4–15.9		
Male, N (%) Female, N (%)	38 (55.9) 30 (44.1)		
Familial inheritance, N (%)	28 (41.2)		
Plexiform neurofibroma, N (%)	22 (32.8)		
Optic pathway glioma, N (%)	11 (16.4)		
Social risk—high, N (%)	14 (20.6)		
Full-Scale IQ, M (SD)	84.6 (12.6)	< .001*	− 1.22 (− 1.53, − 0.90)
Verbal Comprehension Index, M (SD)	88.1 (14.7)	< .001*	− 0.81 (− 1.09, − 0.54)
Visual Spatial Index, M (SD)	87.8 (14.7)	< .001*	− 0.83 (− 1.11, − 0.55)
Fluid Reasoning Index, M (SD)	88.6 (13.5)	< .001*	− 0.85 (− 1.13, − 0.56)
Intellectual disability, N (%)	8 (11.8)		
Word Delay, N (%)	16 (24.6)		
Phrase Delay, N (%)	25 (39.1)		
*Sex normed T-scores, M (SD)*			
SRS-2 Total	75.3 (10.2)	< .001*	2.47 (1.99, 2.95)
SRS-2 Social Communication/Interaction	74.3 (9.5)	< .001*	2.55 (2.05, 3.04)
SRS-2 Restricted/Repetitive Behaviour	74.9 (13.8)	< .001*	1.81 (1.42, 2.20)
Conners Inattention	77.4 (12.5)	< .001*	2.09 (1.58, 2.58)
Conners Hyperactivity–Impulsivity	76.4 (14.3)	< .001*	1.85 (1.38, 2.31)
CBCL Anxiety Problems	63.2 (10.1)	< .001*	1.20 (0.80, 1.60)
CBCL Affective Problems	66.3 (8.9)	< .001*	1.36 (0.95, 1.75)
CBCL Oppositional Defiant Problems	60.9 (10.1)	< .001*	0.98 (0.59, 1.37)

*CBCL* Child Behavior Checklist, *Conners* Conners-Third Edition or Conners ADHD DSM-IV

Rating Scale, *d* Cohen’s *d*, *IQ* intelligence quotient, *M* mean, *CI* confidence interval, *SD* standard

deviation, *SRS-2* Social Responsiveness Scale-Second Edition

^a^N ranges between 64 and 68

*Indicates significant difference from population norms after FDR corrections

### Core features of autism

Mean ADI-R and ADOS-2 algorithm domain scores are presented in Table 2. Approximately half the sample met ADI-R lifetime algorithm cut-offs for the Social, Communication, and RRB domains, and 34% met criteria across all three domains. The ADOS-2 ‘autism spectrum’ cut-off was met by 63% of participants, and 48% were rated as having ‘moderate’ or ‘high’ evidence of autistic behaviours, as indicated by the Overall-CSS. Over half the sample achieved comparison scores consistent with ‘moderate’ or ‘high’ evidence of autistic behaviours for the ADOS-2 domains of Social Affect (55%) and RRB (68%). Further information regarding the overlap between the proportion of participants that fell in the SA-CSS and RRB-CSS severity categories, and the contribution of SA-CSS and RRB-CSS to Overall-CSS can be found in Table S1 (Additional file 1). All ADI-R and ADOS-2 domain scores were comparable for participants with familial versus sporadic NF1 inheritance type (all, *p* > 0.32).

**Table 2:** Mean (95% CI) and proportion meeting cut-offs on ADI-R and ADOS-2 diagnostic algorithms and domains

ADI-R (*N* = 65)	Mean (95% CI)	Met cut-off N (%)				
Social Interaction (Cut-off = 10)	11.7 (9.8, 13.6)	32 (49.2)				
Communication (Cut-off = 8)	9.5 (8.0, 10.9)	36 (55.4)				
RRB (Cut-off = 3)	2.9 (2.4, 3.5)	38 (58.5)				
Met criteria for all three algorithms		22 (33.8)				

			Evidence of autism symptoms based on CSS *N* (%)			
ADOS-2 (*N* = 65)	Mean (95% CI)	Met AS cut-off *N* (%)	No/Minimal (1–2)	Low (3–4)	Moderate (5–7)	High (8–10)
Overall Raw Score (AS Cut-off = 7/8)	8.5 (7.4, 9.7)	41 (63.1)				
Overall-CSS	4.8 (4.2, 5.4)		12 (18.5)	22 (33.8)	20 (30.8)	11 (16.9)
Social Affect-CSS	5.4 (4.8, 6.0)		8 (12.3)	21 (32.3)	21 (32.3)	15 (23.1)
RRB-CSS	4.5 (3.8, 5.2)		21 (32.3)	N/A	37 (56.9)	7 (10.8)

*ADI-R* Autism Diagnostic Interview-Revised, *ADOS-2* Autism Diagnostic Observation Schedule-Second Edition, *AS* autism spectrum, *CI* confidence interval, *CSS* calibrated severity score, *N/A* not applicable as RRB-CSS is not a full 10-point metric (i.e. there are no RRB-CSS of 2–4), *RRB* restricted/repetitive behaviours

*Three participants declined participation in the ADOS-2 and three participants declined participation in the ADI-R

**Fig. 2:**
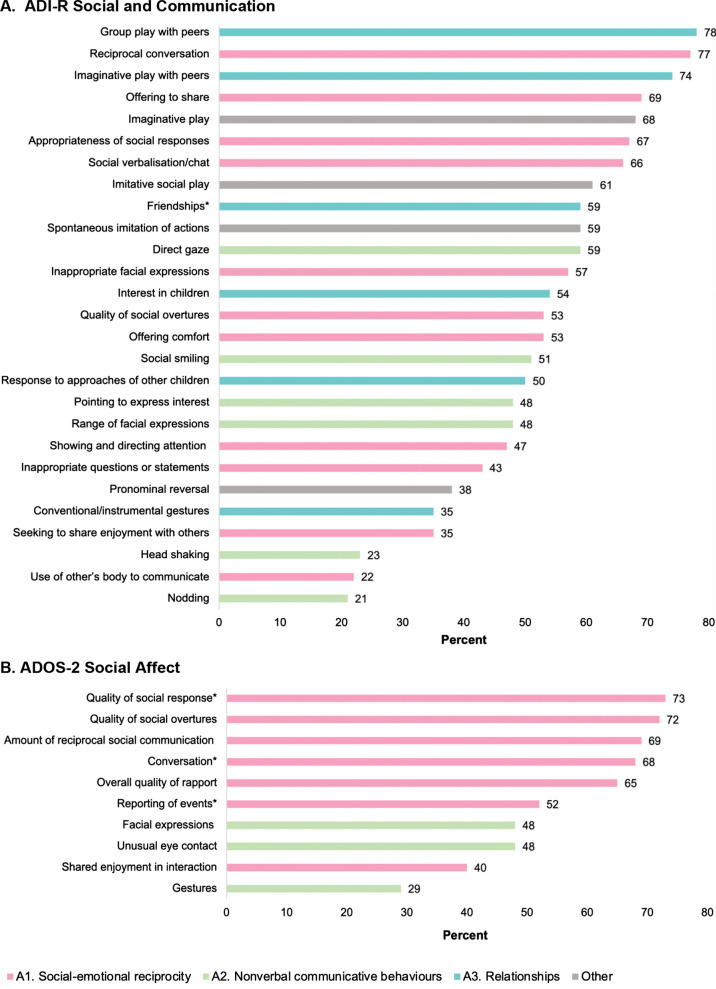
**a** Percentage of sample endorsed with difficulties for items in ADI-R Social and Communication domains, N ranges between 61 and 65, ^*^Friendships item (*N* = 30). **b** Percentage of sample endorsed with difficulties for items in ADOS-2 Social Affect domain (*N* = 65), ^*^item specific to Module 3 (*N* = 56), Note: coloured bars represent DSM-5 diagnostic criteria

**Fig. 3:**
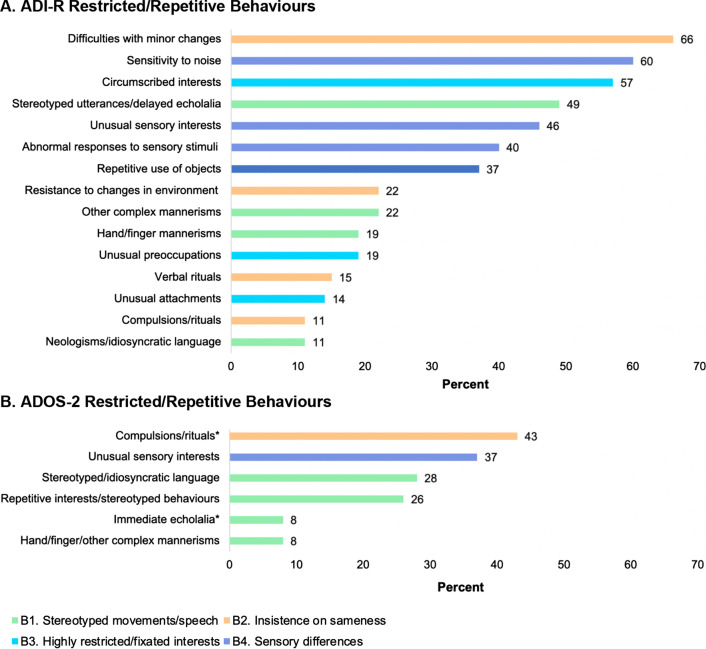
**a** Percentage of sample endorsed with restricted/repetitive behaviours on the ADI-R (*N* = 65). **b** Percentage of sample endorsed with restricted/repetitive behaviours on the ADOS-2 (*N* = 65), ^*^item specific to Module 3 (*N* = 56), Note: coloured bars represent DSM-5 diagnostic criteria

We examined endorsement of autistic behaviours at an item level for the ADI-R lifetime ratings and the ADOS-2. Figures 2 and 3 depict the proportion of participants in which autistic behaviours were endorsed (nonzero codings) in the social communication and RRB domains, respectively. Frequency of symptom severity codings (i.e. 1 versus 2/3) with means and SDs is included as supplementary data (Additional file 1: Tables S2 and S3). For ADI-R social communication behaviours, 59% (16 out of 27 items) were rated as ‘commonly’ impaired (all > 50%, see Fig. 2a). These endorsed items spanned the areas of peer relationships, imaginative play, and reciprocal conversation, as well as use of eye contact and social smiling. Less commonly endorsed social communication impairments were use of nonverbal gestures such as nodding and head shaking, but abnormal pointing was endorsed in almost half the sample. As shown in Fig. 2b, high parent endorsement of social communication difficulties was supported by clinician ratings on the ADOS-2. Particularly pronounced were impairments in social overtures and responses as well as in reciprocal verbal and nonverbal social communication skills. Unusual eye contact and limited facial expressions were clinician-observed in close to one-half of this cohort.

Commonly endorsed RRBs on the ADI-R were difficulties with minor changes, undue sensitivity to noise, and circumscribed interests (all > 50%, see Fig. 3a). Infrequently endorsed behaviours included motor stereotypies, rituals, compulsions, and unusual preoccupations and attachments (all < 25%). No RRBs were coded by clinicians on the ADOS-2 in > 50% of the sample (see Fig. 3b). Compulsions or rituals and unusual sensory interests were, however, observed in 43% and 37% of participants, respectively. Consistent with parent report, there was minimal evidence of motor stereotypies on the ADOS-2 (8%).

### Associations between autistic behaviours and child characteristics

Correlations between domain-level scores on the ADI-R and ADOS-2, SRS-2 total T-score, and child characteristics in our NF1 cohort are presented in Table 3. First examining relationships via the autism-specific diagnostic tools, the presence of Word Delay (*r* = 0.40, *p* < 0.001) and Phrase Delay (*r* = 0.43, *p* < 0.001) was significantly but weakly associated with higher levels of parent-reported lifetime communication impairments on the ADI-R. Univariate linear regression revealed that a positive history of Phrase Delay significantly predicted higher lifetime scores on Communication-ADI (R^2^ = 0.16, *p* = 0.001; 16% of variance explained). We only employed Phrase Delay for regression analysis as this variable showed larger associations with ADI-R scores than Word Delay, and Word Delay was highly correlated with Phrase Delay (*rho* = 0.615, *p* < 0.001). A trend was identified for a relationship between Phrase Delay and Social-ADI (*r* = 0.31, *p* = 0.012) and SA-CSS (*rho* = 0.32, *p* = 0.012). Further, we detected a trend-level association between verbal intellect (VCI) and current ADI-R Communication (*r* = − 0.27, *p* = 0.035) and ADOS-2 Social Affect domains (*rho* = − 0.28, *p* = 0.023). In contrast, RRB domains were not related to early language delay for either autism-specific measure (all; *p* > 0.05).

**Table 3:** Current ADI-R and ADOS-2 domain and SRS-2 correlations with child characteristics

Child characteristics	ADI-R (*N* = 65)^b^			ADOS-2 (*N* = 65)^b^		SRS-2 (*N* = 68)^c^
	Social	Comm	RRB	SA-CSS	RRB-CSS	Total T-score
Word Delay^a^	.28	.40*	.13	.22	− .01	− .10
Phrase Delay^a^	.31	.43**	.06	.32	− .09	− .15
Full-Scale IQ	− .03	− .17	.06	− .05	.01	− .11
Verbal Comprehension Index	− .16	− .27	.02	− .28	− .04	− .12
Visual Spatial Index	.05	.02	.11	.22	.09	.01
Fluid Reasoning Index	− .01	− .04	.12	.22	.19	− .09
Conners Inattention	.20	.20	.11	.14	.10	.42**
Conners Hyperactivity/Impulsivity	.36*	.28	.29	.05	.12	.48**
CBCL Anxiety Problems	.06	− .15	.09	− .15	.25	.34*
CBCL Affective Problems	.12	.05	.23	− .17	.04	.25
CBCL Oppositional Defiant Problems	.03	− .04	− .01	.02	.11	.36*
Social Risk	− .10	− .07	− .02	− .09	.11	.07

*ADI-R* Autism Diagnostic Interview-Revised, *ADOS-2* Autism Diagnostic Observation Scale-Second Edition, *CBCL*

Child Behavior Checklist, *Comm* communication, *Conners* Conners-Third Edition or Conners ADHD DSM-IV Rating

Scale, *CSS* calibrated severity score, *IQ* intelligence quotient, *RRB* restricted/repetitive behaviours, *SA* Social Affect, *SRS-2* Social Responsiveness Scale-Second Edition

^a^ADI-R lifetime scores used to calculate correlation coefficients, ^b^*N* ranges between 62 and 65, ^c^*N* ranges between 64 and 68

*p < .01, **p < .001

Next, we examined the relationship between autistic and ADHD behaviours. We found a weak positive correlation between hyperactive/impulsive ADHD symptoms and Social-ADI (r = 0.36, *p* = 0.003) and trend-level associations with the ADI-R Communication (r = 0.28, *p* = 0.037) and RRB domain scores (r = 0.29, *p* = 0.018). No significant relationships were evident for any of the ADI-R domains with inattentive ADHD symptoms (all; *p* > 0.05). No associations were apparent between either inattentive or hyperactive/impulsive ADHD symptoms and autistic behaviours observed on the ADOS-2 (all; *p* > 0.05). Emotional and behavioural difficulties, as assessed with the CBCL, demonstrated negligible correlations with autistic behaviours (all; *p* > 0.05).

We observed a somewhat different pattern when examining associations between autistic behaviours, as indexed by the SRS-2, and child clinical characteristics. Significant weak-to-moderate correlations between SRS-2 total scores and inattentive (*r* = 0.42, *p* < 0.001) and hyperactive/impulsive ADHD symptom scores (*r* = 0.48, *p* < 0.001) were evident. We further detected significant but weak positive associations between SRS-2 scores, and Anxiety (*r* = 0.34, *p* = 0.006) and Oppositional Defiant Problems on the CBCL (*r* = 0.36, *p* = 0.003). There was a trend for a positive relationship between the SRS-2 and Affective Problems (*r* = 0.25, *p* = 0.042).

## Discussion

The current study set out to characterise the autistic behaviour profile in children with NF1 using item and domain-level analyses of data obtained from the ADI-R and ADOS-2. We examined phenotypic presentation in a screen-positive NF1 sample, selected on the basis of scoring in the clinical range on the SRS-2. This provided a cohort with raised levels of autistic behaviours who may or may not meet threshold for autism diagnosis. To address the debate around whether features of autism in NF1 are driven by the broader NF1 neurodevelopmental phenotype, we also explored associations between autistic behaviours and cognitive, language, and behavioural comorbidities in our NF1 cohort. This study is the first to perform detailed global and local analyses of autistic behaviours with both gold standard measures of the ADI-R and ADOS-2, and its findings raise important points regarding the autistic phenotype in children with NF1.

First, at a global level of analysis, we found that 63% of our screen-positive cohort met the standardised cut-off for ‘autism spectrum’ on the ADOS-2 and 34% exceeded all three algorithm cut-offs on the ADI-R. It is likely that the ADI-R’s more stringent ‘threshold’ for a classification of autism contributed to the substantially smaller proportion of children exceeding all three cut-offs on this measure (including RRBs), as compared with the single cut-off provided by the ADOS-2 that does not require the establishment of RRBs. While it needs to be emphasised that meeting cut-off scores does *not* equate to an autism diagnosis [70, 71], these findings are consistent with prior studies that have demonstrated significantly elevated autistic behaviours in children with NF1 [23, 28, 29, 37].

Second, item-level analyses of social communication symptoms suggest a phenotype that is broadly consistent with idiopathic autism. We initially grouped individual items from these measures as per DSM-5 diagnostic criteria [1]. Items evaluating the A1 criterion of ‘Deficits in social-emotional reciprocity’ were frequently observed by clinicians and reported by parents, including quality of social overtures and responses, reciprocal social communication, and social smiling. In terms of the A2 criterion of ‘Deficits in nonverbal communicative behaviours’, abnormal use of eye contact, facial expressions, and pointing were all reported in a substantial proportion of participants. Interestingly, abnormalities in conventional gestures (e.g. clapping, nodding) were endorsed in fewer children by both clinicians and parents. Criterion A3 of ‘Deficits in developing, maintaining, and understanding relationships’ was only addressed by the ADI-R. All items applying to this criterion including interest in children, friendships, and imaginative play with peers were commonly reported as atypical, highlighting the marked peer relationship difficulties experienced by children with NF1 [26, 72]. These findings are broadly consistent with prior research [35, 37] but extend the current NF1 literature by reporting novel ADI-R item-level data. While two previous studies have reported mean scores for ADOS-2 item-level data [35, 37], these pertain to children with a research classification of autism and cannot be directly compared to our results. It is worth highlighting, however, that Garg and colleagues’ [35] conclusion of better eye contact in children with NF1 is at odds with our results that suggest similarly impaired eye contact in our screen-positive cohort to ADOS-2 normative data for children with ‘autism spectrum’ [33]. While the reason for this discrepancy is unclear, our findings are consistent with eye-tracking studies that have reported atypical eye gaze towards the face and other socially relevant information in NF1 [73, 74]. We also note differences between our sample and the cohort in Garg et al., which was diagnosed with autism based on SRS-2 and ADOS-2 cut-offs and thus exhibited at least a comparable if not more severe overall level of autistic behaviours as our screen-positive sample. Verbal IQ of the Garg et al. sample was 6 points higher than the current study’s NF1 sample; however, this difference is not a large, and we did not find evidence of an association between eye contact and VCI. As such, this between-study discrepancy is most appropriately viewed as a cohort effect and future studies are needed to help determine the weight of evidence for the prominence of atypical eye contact in children with NF1-related autism.

Third, there is evidence of a unique RRB phenotype in children with NF1. Regarding the DSM-5 B1 criterion of ‘Stereotyped or repetitive motor movements, use of objects, or speech’, motor mannerisms were infrequently endorsed on either the ADI-R or ADOS-2 (i.e. less than 23% of the sample). These findings are congruent with previous observations in children with NF1 [23, 35, 36]. Some inconsistencies were evident when examining frequency of endorsement on the stereotyped speech component of the B1 criterion across the two instruments and within the ADI-R. The ADI-R Neologisms/Idiosyncratic Language item, as defined by the use of non-words and unusual words or phrases, was rarely endorsed in our sample (11%), whereas Stereotyped Utterances/Delayed Echolalia was more commonly reported by parents (49%). Since both items may be endorsed because of a variety of behaviours, the exact nature of language abnormalities is difficult to gauge from this information. The ADOS-2 Stereotyped/Idiosyncratic Language item, endorsed in 28% of our sample, similarly incorporates a broad range of language features including echolalia, neologisms, across-person pronoun errors, repetitive utterances, and idiosyncratic or stereotyped speech. Future research is warranted to more precisely characterise the types of language abnormalities considered to constitute RRBs in children with NF1, using dedicated language measures that can better examine their relationship with structural as well as pragmatic language skills. Under the B2 criterion of ‘Excessive adherence to routines, ritualised patterns, or resistance to change’, difficulties with minor changes were commonly parent-reported (66%), with less evidence of resistance to trivial changes in the environment (22%). Interestingly, divergent patterns emerged regarding the presence of rituals and compulsions on the ADI-R (11%) and ADOS-2 (43%). Further work will be required to clarify causes of this difference, e.g. whether the interpretation of behaviours required for endorsement of compulsions and rituals varies for caregivers versus clinicians. Differential endorsement of items comprising the ‘Highly restricted, fixated interests’ B3 criterion was also observed, with common parent report of circumscribed interests (57%), but infrequent endorsement of unusual preoccupations (19%) and unusual attachment to objects (14%). Regarding the B4 criterion of atypical sensory processing, a substantial proportion of our sample were parent-reported to display sensory aversions, i.e. sensitivity to noise (60%) and abnormal responses to sensory stimuli (40%). Further, unusual sensory interests were endorsed by parents and clinicians in 46% and in 37% of our sample, respectively.

Fourth, RRBs in NF1 appear to be dominated by an insistence on sameness. Since DSM-5 categorisations of RRBs are not empirically derived [2], another way of considering these behaviours is to examine their relationship to distinct RRB subcategories identified in idiopathic autism. Factor analytic studies have supported the presence of two dimensions, ‘repetitive sensory motor’ (RSM) and ‘insistence on sameness’ (IS) behaviours. RSM behaviours comprise motor mannerisms, repetitive use of objects, and sensory-seeking behaviours, which tend to negatively correlate with age and IQ in idiopathic autism samples [75–77]. In turn, IS behaviours include resistance to change, compulsions and rituals, circumscribed interests, and sensitivity to noise [75–78]. In this context, it is notable that the key RRBs in our cohort were all IS behaviours, driven by difficulties with minor changes, circumscribed interests, and sensitivity to noise. This novel finding has intriguing implications for a distinct profile of autistic behaviours in NF1. We could speculate that this pattern of more pronounced IS behaviours, which have been conceptualised as ‘higher-order’ RRBs [77], may relate to the relatively higher and truncated IQ in NF1 [48], as compared to the greater prevalence of severe intellectual impairments in idiopathic autism [5]. Indeed, circumscribed interests, together with compulsions and rituals, have been found to positively correlate with IQ in autistic children [79], although some studies have shown no association or only small positive associations between IQ and IS behaviours [75–77]. It is also interesting to consider how common NF1 characteristics such as executive deficits [80] and anxiety [81] may affect the expression of IS behaviours in NF1, since these constructs conceptually converge and have overlapping symptom presentations (e.g. cognitive/behavioural inflexibility). Executive deficits and anxiety have been suggested to relate to IS characteristics in idiopathic autism [82, 83], but such potentially informative associations are yet to be explored in children with NF1. Moving forward, subsequent studies should investigate whether the IS and RSM dimensions found in idiopathic autism also emerge in NF1, and which specific behaviours cluster together in these, or perhaps different factors.

Fifth, our findings provided convincing evidence that the ADOS-2 and ADI-R captured distinct autistic behaviours in our cohort that were not attributable to the common NF1 comorbidities evaluated in this study. We observed weak positive associations between parent-reported hyperactive/impulsive ADHD symptoms and autistic behaviours on the ADI-R, but not on the ADOS-2. Moreover, neither ADI-R nor ADOS-2 ratings, in any domain, were related to parent-reported inattentive ADHD, internalising, and oppositional defiant symptoms. In contrast, we detected weak-to-moderate positive associations between SRS-2 scores and ratings from measures evaluating ADHD, anxiety, and oppositional defiant symptoms in our sample. These relatively stronger correlations, as compared with the ADI-R and ADOS-2, are likely due to common rater bias between the SRS-2 and the other parent rating scales, as well as reduced specificity of the SRS-2 in children with comorbid childhood neurodevelopmental disorders such as ADHD, anxiety, and high levels of challenging behaviours [50, 84, 85]. Our findings are also broadly consistent with the moderate-to-high positive associations between SRS-2 and CBCL scores reported by Morotti et al. [41], which were partly used to contend that ‘autism-like’ behaviours in their NF1 sample were better explained by ADHD and internalising symptoms. While we acknowledge concerns regarding the specificity of the SRS-2 in differentiating autism from other neurodevelopmental disorders (e.g. ADHD, anxiety) [50, 84, 85], it is important to recognise that a significant relationship between autistic and non-autistic behaviours does not ‘explain away’ autism [43]. Indeed, prior research has demonstrated substantial correlations between features of autism and ADHD in NF1 [21, 24–26], and it is likely that these associations reflect the tendency for these characteristics to coexist in NF1, as is the case in idiopathic autism [7].

We further detected weak-to-moderate relationships between early language proficiency and verbal intellect, and social communication difficulties on the ADI-R and ADOS-2. Again, some degree of association between these constructs would be anticipated given that language and verbal abilities have been shown to affect social communication behaviours in children with and without autism [86, 87]. It is, however, worth noting the weak and non-significant relationship between verbal intellect and current communication scores on the ADI-R in our cohort. This finding may partly be due to several items in the ADI-R Communication domain assessing nonverbal communicative behaviours that are not contingent on verbal abilities (e.g. gestures and spontaneous imitation of actions) and pragmatic language deficits impairing social communication even in the context of intact structural language and verbal skills [2, 88]. At present, the nature of these observed associations between core autism features and other clinical characteristics of NF1 are unclear, and longitudinal studies will be required to determine their causal relationships.

Our identification of a distinct autistic phenotype in children with NF1 may aid clinicians’ awareness of the presentation of autism in this population and facilitate more accurate and timely recognition of the disorder. IS behaviours such as restricted patterns of interest and ‘just right’ behaviours are relatively common features of typical early development [89] that may be less likely to be recognised as manifestations of autism than stereotyped and unusual behaviours. In this context, social communication difficulties in children with NF1, although present, may not be sufficiently pronounced in the relatively undemanding and well scaffolded home and early childcare settings to raise a flag for autism. More precise characterisation of the behaviour profile of autism in children with NF1 may also be used to help guide the design and implementation of early therapeutic interventions to ameliorate prominent and debilitating characteristics of autism in NF1. While autism-specific interventions are yet to be formally evaluated in children with NF1, positive responses to treatment have been shown to be highly variable amongst individuals with idiopathic autism [90–93]. Children with NF1-related autism likely represent a more circumscribed population in which to assess the efficacy of specific components of early behavioural intervention programs. Indeed, given evidence in the idiopathic literature that higher child cognitive and language skills and less severe autistic behaviours are associated with better treatment responses [94], we would anticipate that children with NF1 have the potential to gain significant benefits from appropriately designed interventions.

Taken together, our findings support the elevation of autistic behaviours in a significant subset of children with NF1 and underscore the importance of considering potentially confounding factors to guide interpretation of ‘autistic-like’ behaviours in NF1. Since salient features of the NF1 clinical phenotype overlap with classical autistic behaviours, scores on autism measures may be spuriously increased and not validly reflect autistic behaviours or diagnosis in the condition. Although the ADOS-2 and ADI-R are clinician-rated tools with high sensitivity and specificity for autism [32], scores on these measures have also been shown to be inflated by cognitive impairments and behavioural disorders not specific to autism [51, 95]. It is important for future research to address these issues. In particular, it is critical that research classifications of autism in NF1 are systematically compared to diagnoses ascertained through best estimate multidisciplinary clinical judgement [70, 71], taking into account the influence of non-autism-specific factors on instrument scores and diagnostic decisions. Such analyses are key to refining our use of autism diagnostic tools in NF1 and will be essential to progress our understanding of the autistic phenotype in children with NF1.

### Limitations

Limitations of the current study include the following. First, the absence of genetic data in this cohort precludes examination of potentially informative associations between autistic behaviours and sequence variants within *NF1, NF1* microdeletions, and the impact of common genetic variants not related to NF1. Given strong evidence for more severe phenotypic presentations in individuals with *NF1* microdeletions compared to those with intragenic mutations [96], and the likely influence of common genetic variants [97], larger research studies dissecting these relationships will be an important avenue for advancing our understanding of genomic and neurobiological risk for autism in NF1. Second, our screen-positive cohort of children with NF1 represents a subset of children selected on the basis of scores in the clinical range on a screening questionnaire and thus is not representative of all children with NF1. However, given the high sensitivity of the SRS-2 for autism [46] and that many children in our sample with elevated SRS-2 scores exhibited minimal evidence of autistic behaviours on the ADOS-2 and ADI-R, it is unlikely that our screen-positive cohort failed to capture many children with significant features of autism. Third, our findings regarding the profile of autistic behaviours in NF1 were derived from a screen-positive sample, rather than children with a diagnosis of autism. This sample was chosen for investigation due to the dimensional nature of autistic behaviours. Efforts are currently underway by our research group to provide more specific conclusions regarding symptom presentation in children with a clinical diagnosis of autism. Fourth, we acknowledge that our characterisation of the autistic phenotype in NF1 was dependent on the specific behaviours indexed by the ADI-R and ADOS-2; future studies utilising different measures may identify additional autistic behaviours of relevance. Finally, although the ADI-R is constructed to minimise recall bias by eliciting concrete examples of behaviours from parents, we cannot exclude the possibility of retrospective bias affecting lifetime ratings on this measure [98]. The field would benefit from longitudinal designs to ascertain the developmental course of autistic behaviours and their relationship with other neurodevelopmental comorbidities in children with NF1.

## Conclusions

In summary, this study characterises the core features of autism in children with NF1, with findings suggestive of a distinct autistic phenotype that has not been previously reported. Social communication behaviours appear to parallel those found in idiopathic autism [99]. Restricted and repetitive behaviours, however, seem to be characterised by more pronounced ‘insistence on sameness’ behaviours such as circumscribed interests, difficulties with minor changes, and sensitivity to noise, with little evidence of motor stereotypies, and unusual interests or attachments. Importantly, our examination of the relationship between autistic behaviours and common NF1 comorbidities indicates that the scores derived from gold standard autism instruments genuinely reflect features of autism in our NF1 cohort, with the caveat that these do not necessarily reflect an autism diagnosis. Our novel characterisation of the autistic phenotype in NF1 has important clinical and research implications. Early recognition of autism is clearly essential for optimising children’s long-term outcomes [100], and a clearer understanding of the autistic profile in NF1 will facilitate the development of screening tools that are more sensitive and specific to detecting autism in this population. This knowledge will also help tailor evidence-based interventions to target the more impairing features of autism in children with NF1. In addition, precise delineation of core autistic behaviours is a crucial step for the establishment of genotype–phenotype associations in NF1 that holds promise for advancing our understanding of the causal mechanisms of autism. Future studies employing larger NF1 cohorts with genetic testing will be required to investigate potential links between *NF1* mutation types and the autistic phenotype in children with NF1. Subsequent research should also carefully examine similarities and differences in the autistic phenotype between children with NF1 and a clinical diagnosis of autism and children with autism from the general population with comparable cognitive abilities.

## Supplementary Information

**Additional file 1**. **Table S1**. Descriptive data for the larger NF1 cohort and by SRS-2 cut-off (*N* = 152). **Table S2**. Number and percent of sample rated with ADOS-2 SA-CSS, RRB-CSS, and Overall-CSS severity levels. **Table S3**. Percent endorsement 1 vs. 2/3 codings and mean (SD) of ADI-R lifetime items and subscales. **Table S4**. Percent endorsement 1 vs. 2/3 codings and mean (SD) of ADOS-2 items.

## Data Availability

The data set generated and analysed during the current study is available from the corresponding author on reasonable request.

## Abbreviations

ADHD: Attention Deficit Hyperactivity Disorder;
ADI-R: Autism Diagnostic Interview-Revised
ADOS-2: Autism Diagnostic Observation Schedule-Second Edition
ASD: Autism spectrum disorder
CI: Confidence interval
Communication-ADI: Autism Diagnostic Interview-Revised Communication domain
CADS: Conners ADHD DSM-IV Rating Scale
CSS: Calibrated severity score
DSM: Diagnostic and Statistical Manual of Mental Disorders
FSIQ: Full-scale intelligence quotient
GTP: Guanosine triphosphate
IS: Insistence on sameness
MAPK: Mitogen-activated protein kinase
M: Mean
mTOR: Mechanistic target of rapamycin
Overall-CSS: Overall calibrated severity score
NF1: Neurofibromatosis type 1
Ras: Rat sarcoma
RRB: Restricted/repetitive behaviours
RRB-ADI: Autism Diagnostic Interview-Revised Restricted/repetitive behaviours domain
RRB-CSS: Restricted/repetitive behaviours calibrated severity score
RSM: Repetitive sensory motor
SA: Social affect
SA-CSS: Social affect calibrated severity score
SD: Standard deviation
Social-ADI: Autism Diagnostic Interview-Revised Social domain
SRS-2: Social Responsiveness Scale-Second Edition
VCI: Verbal Comprehension Index
WISC-V: Wechsler Intelligence Scale for Children-Fifth Edition
WPPSI-IV: Wechsler Preschool and Primary Scale of Intelligence-Fourth Edition
