# Supplementary material for: Delineating the autistic phenotype in children with neurofibromatosis type 1
Source: Mol Autism. 2022 Jan 4;13:3. doi: 10.1186/s13229-021-00481-3 (PMC8729013; doi:10.1186/s13229-021-00481-3)
Supplement: Supplementary file 1 — Additional file 1. Table S1. Descriptive data for the larger NF1 cohort and by SRS-2 cut-off (N = 152). Table S2. Number and percent of sample rated with ADOS-2 SA-CSS, RRB-CSS, and Overall-CSS severity levels. Table S3. Percent endorsement 1 vs. 2/3 codings and mean (SD) of ADI-R lifetime items and subscales. Table S4. Percent endorsement 1 vs. 2/3 codings and mean (SD) of ADOS-2 items. [file 13229_2021_481_MOESM1_ESM.docx]

**Table S1** Descriptive data for the larger NF1 cohort and by SRS-2 cut-off (N = 152)

| **NF1 Characteristics** | **Total** | | **SRS-2 ≥ 60^a^** | | **SRS-2 < 60** | | **Group comparisons** | | |
| --- | --- | --- | --- | --- | --- | --- | --- | --- | --- |
|  | **N** |  | **N** |  | **N** |  | ***t / χ^2^*** | ***p*** | ***d / φ^c^*** |
| Age in years, M (SD)  Range | 152 | 8.3 (3.4)  3.0 - 15.9 | 68 | 9.0 (3.4)  3.4 - 15.9 | 75 | 7.8 (3.4)  3.0 - 15.3 | 2.04 | .043* | 0.34 |
| Male, N (%) | 152 | 86 (56.6) | 68 | 38 (55.9) | 75 | 43 (57.3) | 0.00 | .995 | -0.02 |
| Female, N (%) | 152 | 88 (43.4) | 68 | 30 (44.1) | 75 | 32 (42.7) |  |  |  |
| Familial inheritance, N (%) | 151 | 59 (39.3) | 67 | 28 (41.2) | 75 | 26 (34.7) | 0.49 | .484 | -0.07 |
| Plexiform neurofibroma, N (%) | 151 | 36 (23.8) | 67 | 22 (32.8) | 74 | 13 (17.6) | 3.61 | .057 | 0.18 |
| Optic pathway glioma, N (%) | 151 | 28 (18.5) | 67 | 11 (16.4) | 75 | 16 (21.3) | 0.28 | .595 | -0.06 |
| Social risk - high, N (%) | 147 | 22 (15.0) | 65 | 14 (21.9) | 74 | 7 (9.5) | 3.94 | .059 | -0.17 |
| Full Scale IQ, M (SD) | 152 | 88.6 (12.8) | 68 | 84.6 (12.6) | 75 | 92.7 (11.3) | -4.05 | <.001* | -0.68 |
| Verbal Comprehension Index, M (SD) | 151 | 93.6 (14.6) | 68 | 88.1 (14.7) | 74 | 98.6 (11.7) | -4.73 | <.001* | -0.79 |
| Visual Spatial Index, M (SD) | 150 | 88.0 (12.4) | 67 | 87.8 (14.7) | 74 | 89.1 (10.1) | -0.61 | .553 | -0.10 |
| Fluid Reasoning Index, M (SD) | 137 | 90.8 (13.8) | 65 | 88.6 (13.5) | 64 | 93.6 (13.7) | -2.09 | .038* | -0.37 |
| Intellectual disability, N (%)^b^ | 152 | 12 (7.9) | 68 | 8 (11.8) | 75 | 2 (2.7) | 0.24 | 1.00^d^ | -0.15 |
| Word Delay, N (%) | N/A | | 65 | 16 (24.6) | N/A | |  |  |  |
| Phrase Delay, N (%) | N/A | | 64 | 25 (39.1) | N/A | |  |  |  |
| *Sex normed T-scores, M (SD)* |  |  |  |  |  |  |  |  |  |
| SRS-2 Total | 152 | 62.4 (14.8) | 68 | 75.3 (10.2) | 75 | 49.9 (5.7) | 18.58 | <.001* | -3.11 |
| SRS-2 SCI | 151 | 61.7 (14.4) | 67 | 74.3 (9.5) | 74 | 49.5 (6.0) | 18.70 | <.001* | -3.15 |
| SRS-2 RRB | 151 | 62.4 (15.8) | 67 | 74.9 (13.8) | 75 | 50.5 (6.4) | 13.74 | <.001* | -2.31 |
| Conners Inattention | 149 | 67.9 (16.5) | 68 | 77.4 (12.5) | 72 | 58.5 (15.0) | 5.05 | <.001* | -1.06 |
| Conners Hyperactivity-Impulsivity | 149 | 65.7 (17.6) | 68 | 76.4 (14.3) | 72 | 55.6 (14.5) | 6.69 | <.001* | -1.40 |
| CBCL Anxiety Problems | 143 | 58.4 (9.4) | 66 | 63.2 (10.1) | 72 | 53.5 (5.7) | 6.81 | <.001* | -1.19 |
| CBCL Affective Problems | 144 | 61.1 (9.7) | 66 | 66.3 (8.8) | 73 | 55.8 (7.5) | 7.57 | <.001* | -1.29 |
| CBCL Oppositional Defiant Problems | 143 | 57.2 (9.0) | 66 | 61.0 (10.1) | 72 | 53.1 (4.7) | 5.87 | <.001* | -1.03 |

*CBCL* Child Behavior Checklist, *Conners* Conners-Third Edition or Conners ADHD DSM-IV Rating Scale, *d* Cohen’s *d*, *IQ* intelligence quotient, *M* mean, *N/A* not administered, *φ* phi, *RRB* restricted/repetitive behaviours, *SCI* social communication/interaction, *SD* standard deviation, *SRS-2* Social Responsiveness Scale-Second Edition

^a^ Nine participants did not complete either the Autism Diagnostic Interview-Revised or the Autism Diagnostic Observation Schedule-Second Edition and were excluded from this sample

^b^ Participants with a clinical diagnosis of intellectual disability

^c^ Negative effect sizes express greater impairment in SRS-2 screen positive sample compared with screen negative sample for cognitive/language and parent questionnaire comparisons

^d^ Fisher’s Exact Probability Test used as < 5 expected frequency in each cell

* indicates significant difference between groups after FDR

**Table S2** Number and percent of sample rated with ADOS-2 SA-CSS, RRB-CSS, and Overall-CSS severity levels

|  | **ADOS-2 severity level of autism-related behaviours (N = 62)** | | | | | | | | |
| --- | --- | --- | --- | --- | --- | --- | --- | --- | --- |
|  |  | **Social Affect CSS** | | | | **Overall CSS** | | | |
|  | **N (%)** | **Minimal** | **Low** | **Moderate** | **High** | **Minimal** | **Low** | **Moderate** | **High** |
| **RRB**  **CSS** | **Minimal** | 4  6.5% | 8  12.9% | 4  6.5% | 4  6.5% | 7  11.3% | 8  12.9% | 4  6.5% | 1  1.6% |
|  | **Moderate** | 2  3.2% | 10  16.1% | 14  22.6% | 10  16.1% | 2  3.2% | 13  21.0% | 14  22.6% | 7  11.3% |
|  | **High** | 0  0% | 2  3.2% | 3  4.8% | 1  1.6% | 0  0% | 1  1.6% | 2  3.2% | 3  4.8% |
| **Overall**  **CSS** | **Minimal** | 6  9.7% | 3  4.8% | 0  0% | 0  0% |  |  |  |  |
|  | **Low** | 0  0% | 16  25.8% | 6  9.7% | 0  0% |  |  |  |  |
|  | **Moderate** | 0  0% | 1  1.6% | 13  21.0% | 6  9.7% |  |  |  |  |
|  | **High** | 0  0% | 0  0% | 2  3.2% | 9  14.5% |  |  |  |  |

*ADOS-2* Autism Diagnostic Observation Schedule-Second Edition, *CSS* calibrated severity score, *RRB* restricted/repetitive behaviours

Note: Since RRB-CSS is not a full 10-point severity metric there is no ‘Low’ RRB-CSS

**Table S3** Percent endorsement 1 vs. 2/3 codings and mean (SD) of ADI-R lifetime items and subscales

| **ADI-R algorithm items (N = 65)^a^** | **% Severity coding**  **1 2/3** | | **Mean (SD)** |
| --- | --- | --- | --- |
| *Failure to use nonverbal behaviours to regulate social interaction* | | | 2.31 (1.91) |
| Direct gaze | 29.2 | 29.2 | 0.89 (0.87) |
| Social smiling | 29.2 | 21.5 | 0.78 (0.93) |
| Range of facial expressions | 25.4 | 22.2 | 0.70 (0.82) |
| *Failure to develop peer relationships* | | | 3.65 (2.36) |
| Imaginative play with peers | 36.5 | 38.1 | 1.25 (0.98) |
| Interest in children | 22.2 | 31.7 | 1.03 (1.15) |
| Response to approaches of other children | 37.5 | 12.5 | 0.67 (0.82) |
| Group play with peers | 39.7 | 38.1 | 1.30 (0.98) |
| Friendships (>10.0 years) | 27.6 | 31.0 | 0.97 (0.98) |
| *Lack of shared enjoyment* | | | 2.29 (2.03) |
| Showing and directing attention | 25.0 | 21.9 | 0.77 (0.97) |
| Offering to share | 26.6 | 42.2 | 1.22 (1.11) |
| Seeking to share enjoyment with others | 18.5 | 16.9 | 0.52 (0.77) |
| *Lack of socioemotional reciprocity* | | | 3.46 (2.61) |
| Use of other’s body to communicate | 15.6 | 6.3 | 0.30 (0.63) |
| Offering comfort | 23.8 | 28.6 | 0.98 (1.14) |
| Quality of social overtures | 32.8 | 20.3 | 0.80 (0.91) |
| Inappropriate facial expressions | 36.9 | 20.0 | 0.77 (0.77) |
| Appropriateness of social responses | 34.4 | 32.8 | 1.08 (0.95) |
| *Lack/delay in spoken language and failure to compensate through gesture* | | | 1.86 (2.14) |
| Pointing to express interest | 33.9 | 14.5 | 0.63 (0.73) |
| Nodding | 9.7 | 11.3 | 0.32 (0.67) |
| Head shaking | 11.5 | 11.5 | 0.34 (0.68) |
| Conventional / instrumental gestures | 9.2 | 26.2 | 0.65 (0.94) |
| *Lack of varied spontaneous make-believe or social imitative play* | | | 3.03 (1.98) |
| Spontaneous imitation of actions | 10.8 | 47.7 | 0.65 (0.94) |
| Imaginative play | 24.6 | 43.1 | 3.03 (1.98) |
| Imitative social play | 34.4 | 26.6 | 1.28 (1.22) |
| *Relative failure to initiate or sustain conversational interchange* | | | 2.46 (1.51) |
| Social verbalisation / chat | 21.5 | 44.6 | 1.11 (0.89) |
| Reciprocal conversation | 18.5 | 58.5 | 1.38 (0.88) |
| *Stereotyped, repetitive or idiosyncratic speech* | | | 2.14 (1.78) |
| Stereotyped utterances and delayed echolalia | 32.3 | 16.9 | 0.69 (0.83) |
| Inappropriate questions or statements | 16.9 | 26.2 | 0.69 (0.87) |
| Pronominal reversal | 7.8 | 29.7 | 0.77 (1.08) |
| Neologisms and idiosyncratic language | 6.2 | 4.6 | 0.15 (0.48) |
| *Encompassing preoccupation or circumscribed interest* | | | 1.14 (1.04) |
| Unusual preoccupations | 12.3 | 6.2 | 0.22 (0.55) |
| Circumscribed interests | 24.6 | 32.3 | 0.18 (0.58) |
| *Apparently compulsive adherence to non-functional routines or rituals* | | | 0.42 (0.90) |
| Verbal rituals | 9.2 | 6.2 | 0.22 (0.55) |
| Compulsions and rituals | 4.6 | 6.2 | 0.18 (0.58) |
| *Stereotyped and repetitive motor mannerisms* | | | 0.52 (0.79) |
| Hand and finger mannerisms | 7.7 | 10.8 | 0.31 (0.71) |
| Other complex mannerisms | 9.2 | 12.3 | 0.38 (0.82) |
| *Preoccupation with parts of objects or non-functional elements of material* | | | 0.94 (0.83) |
| Repetitive use of objects or parts of objects | 13.8 | 23.1 | 0.71 (1.06) |
| Unusual sensory interests | 36.9 | 9.2 | 0.55 (0.66) |
| **ADI-R non-algorithm RRB items** | | |  |
| Undue general sensitivity to noise | 21.5 | 38.5 | 1.32 (1.19) |
| Abnormal responses to specific sensory stimuli | 16.9 | 23.1 | 0.69 (0.97) |
| Difficulties with minor changes | 30.8 | 35.4 | 1.17 (1.07) |
| Resistance to trivial changes in the environment | 10.8 | 10.8 | 0.32 (0.66) |
| Unusual attachment to objects | 6.2 | 7.7 | 0.22 (0.57) |

*ADI-R* Autism Diagnostic Interview-Revised, *RRB* restricted/repetitive behaviours, *SD* standard deviation

**^a^** N ranges between 61 and 65. Note: items are organised by ADI-R subscales

**Table S4** Percent endorsement 1 vs. 2/3 codings and mean (SD) of ADOS-2 items

| **ADOS-2 items (N = 65)** | **% Severity coding**  **1 2/3** | | **Mean (SD)** |
| --- | --- | --- | --- |
| Social Affect algorithm items | | |  |
| Gestures | 27.7 | 1.5 | 0.31 (0.50) |
| Conversation^a^ | 46.4 | 21.4 | 0.89 (0.73) |
| Reporting of events^a^ | 33.9 | 14.3 | 0.71 (0.83) |
| Unusual eye contact | N/A | 47.7 | 0.95 (1.11) |
| Facial expressions | 40.0 | 7.7 | 0.55 (0.64) |
| Shared enjoyment in interaction | 29.2 | 10.8 | 0.51 (0.69) |
| Quality of social overtures | 53.8 | 18.5 | 0.91 (0.68) |
| Quality of social response^a^ | 64.3 | 8.9 | 0.84 (0.63) |
| Amount of reciprocal social communication | 53.8 | 15.4 | 0.85 (0.67) |
| Overall quality of rapport | 49.2 | 15.4 | 0.80 (0.69) |
| Restricted/Repetitive Behaviour algorithm items | | |  |
| Stereotyped/idiosyncratic language | 23.1 | 4.6 | 0.32 (0.56) |
| Unusual sensory interest | 21.5 | 15.4 | 0.52 (0.75) |
| Hand & finger & other complex mannerisms | 3.1 | 4.6 | 0.14 (0.53) |
| Repetitive interests/stereotyped behaviours | 20.0 | 6.2 | 0.32 (0.59) |
| Restricted/Repetitive Behaviour non-algorithm items | | |  |
| Immediate echolalia | 6.2 | 1.5 | 0.09 (0.34) |
| Compulsions or rituals^a^ | 39.3 | 3.6 | 0.46 (0.57) |

*ADOS-2* Autism Diagnostic Observation Schedule-Second Edition, *N/A* not applicable as this item is

coded 0 or 2, *RRB* restricted/repetitive behaviours, *SD* standard deviation

*^a^* item only available for participants administered Module 3 (N = 56)
